# Assessment of Knowledge and Attitude Regarding Teledentistry Among Dental Professionals: A Cross-Sectional Study

**DOI:** 10.7759/cureus.55954

**Published:** 2024-03-11

**Authors:** Paranna Sujatha, Aditi A Kanitkar, Savitri Ranjeri, Ankita Annu, Anil Patil, Jyoti Biradar

**Affiliations:** 1 Pediatric and Preventive Dentistry, Bharati Vidyapeeth (Deemed to be University) Dental College and Hospital, Sangli, IND; 2 Prosthodontics, Bharati Vidyapeeth (Deemed to be University) Dental College and Hospital, Sangli, IND; 3 Pediatric Dentistry, HKDET Dental College, Humnabad, IND; 4 Pediatric Dentistry, Bharati Vidyapeeth (Deemed to be University) Dental College and Hospital, Sangli, IND; 5 Oral and Maxillofacial Surgery, Bharati Vidyapeeth (Deemed to be University) Dental College and Hospital, Sangli, IND

**Keywords:** attitude, knowledge, questionnaire survey, dental professionals, teledentistry

## Abstract

Background: Modern technologies have led to the development of new tools, practices, and digital techniques. However, their use in public health to provide adequate oral health facilities to the community is limited. One of the facilities that can help provide better oral health with minimal cost is teledentistry. The application of this approach will reduce inequalities in accessing oral healthcare. Knowledge of the use of teledentistry is of the utmost importance to its practice. Hence, the objective of this cross-sectional study is to assess the knowledge of and attitude regarding teledentistry among dental professionals in the Sangli district of Maharashtra.

Materials and methods: A 24-unit structured online validated questionnaire with six questions regarding participants' sociodemographic information and 18 questions related to their knowledge and attitude toward teledentistry and informed consent forms were circulated via email among 100 dentists, and the responses obtained were analyzed.

Results: Out of 100 responses, 61 showed basic knowledge and a typical attitude toward teledentistry. Urban practitioners were more familiar with teledentistry than rural ones.

Conclusion: This survey concludes that the branch of teledentistry still needs to be studied and publicized at a greater level to accelerate its widespread implementation in dentistry and especially to increase the outreach and time efficiency of dentistry.

## Introduction

Teledentistry is defined as the "provision of real-time and offline dental care such as diagnosis, treatment planning, consulting, and follow-up via electronic transmission from different sites" [1]. The various objectives of teledentistry are to store the patient information and forward it to the appropriate dental specialist for a consultation and treatment plan and remote monitoring, which includes monitoring the patient through periodic photographs, data, and live video conferences [2].

With the uncertainty of the coronavirus disease 2019 (COVID-19) pandemic, teledentistry is being most commonly used to follow social distancing regulations [3]. Minimizing the number of appointments and reducing the number of patients in the clinic help reduce the risk of viral transmission [4]. The importance of teledentistry became realized during the COVID-19 pandemic, whereas beforehand, it was a neglected concept. As the whole world was hit by the COVID-19 pandemic and healthcare professionals applied telecommunication to treat patients, gaining adequate information regarding teledentistry has become quite evident.

Inadequate access to healthcare is a major challenge worldwide [5], and the challenge includes access to oral healthcare, too [6]. The urgent demands generated by the COVID-19 pandemic motivated practitioners to employ ingenious strategies such as telehealth. The COVID-19 pandemic has gained more attention toward the potential uses of telehealth [7-9], including teledentistry [10]. The pandemic required stricter restrictions on elective medical and dental clinic visits because visits were permitted to emergency cases in many countries [10-12]. Therefore, to achieve better implementation of teledentistry, it is important to understand the current state of teledentistry knowledge, beliefs, and practices among dentists. Hence, the objective of this study was to assess the knowledge of and attitudes regarding teledentistry among dental practitioners in the Sangli district of Maharashtra.

## Materials and methods

A cross-sectional questionnaire study was conducted among practicing dental professionals in Sangli district. The sample size calculation was done using the formula Z^2^_1-α/2_×P(1-P)/d^2^, where n is the required sample size, Z1−α/2​ is the critical value of the standard normal distribution corresponding to the desired confidence level (where α is the significance level), P is the estimated proportion of the population, and d is the desired margin of error.

The sample size derived from the formula was 100. Approval to conduct the survey was obtained from the Institutional Ethics Committee of Bharati Vidyapeeth (Deemed to be University) Medical College and Hospital (approval number: BV(DU)MC&H/Sangli/IEC/0-48/21). A total of 175 dentists registered under the Indian Dental Association of Sangli branch in Maharashtra were sent an invitation to participate in the study. The convenience sampling method was used to represent the population and get a sample size of 100 registered dentists.

Data collection

A self-designed, closed-ended questionnaire was prepared and tested for face validity, content validity, and test-retest reliability. A self-designed, validated questionnaire along with informed consent was prepared with the help of Google Documents. The questionnaire consisted of two main parts: Part 1 included sociodemographic information on age, gender, qualification, experience, and the locality of practice. Part 2 was on knowledge and attitude, which included questions on the opinion of the dentist about teledentistry, the consent requirement, doctor-patient relationship, prescription of medication, oral hygiene training, oral health education, financial feasibility, time concerns, acceptance in robotic dentistry, specialty treatment, media used, guidelines, documentation, benefits, and potential shortcomings of teledentistry.

A dental professional was approached via email with an informed consent form before the self-designed questionnaire was sent. Out of 175 registered dental professionals in Sangli, 100 dentists accepted the invitation to participate in the study. However, 39 dentists failed to respond to the questionnaire after three consecutive reminders and were excluded from the study. The data obtained from 61 dentists was then entered into Microsoft Excel and was analyzed using descriptive statistics.

## Results

One hundred and seventy-five registered dental professionals in Sangli accepted the invitation to participate in the study, but only 100 dentists accepted the invitation to participate in the survey. However, out of 100 dentists who had accepted the invitation, 39 dentists failed to respond to the questionnaire after three consecutive reminders and were subsequently excluded. Out of 100 dentists contacted, only 61% of dentists responded with 34 females and 27 males. The data obtained was then entered into Microsoft Excel and was analyzed using descriptive statistics. The most represented age group was 31-40 years old, with 50.8%. Concerning the area of work, 54.1% were both general practitioners and academicians, 26.2% were general practitioners, and 19.7% were academicians. Furthermore, 26% of respondents had more than 10 years of work experience, 22% had 5-10 years of experience, and 13% had less than five years of experience. Also, 34.4% had a Master of Dental Surgery, and 65.6% had a Bachelor of Dental Surgery. Moreover, 85.2% of respondents were practicing in urban settings and the remaining 14.8% in rural areas (Table 1).

**Table 1:** Sociodemographic data of participants BDS: Bachelor of Dental Surgery; MDS: Master of Dental Surgery

Sociodemographic data	Responses	Percentage
Age (in years)	20-30	16.4%
	31-40	50.8%
	41-50	31.1%
	51-60	1.7%
Gender	Male	44%
	Female	56%
Qualification	BDS	65.6%
	MDS	34.4%
Location of practice	Urban	85.2%
	Rural	14.8%
Area of work	General practice	26.2%
	Academics	19.7%
	Both (general practice and academics)	54.1%

**Figure 1:**
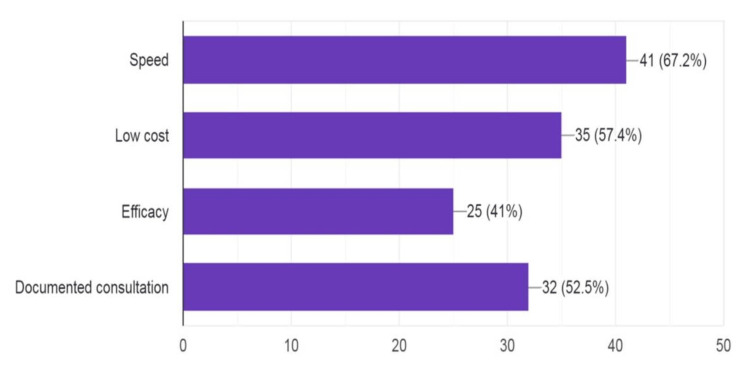
Benefits of internet-based teledentistry

**Figure 2:**
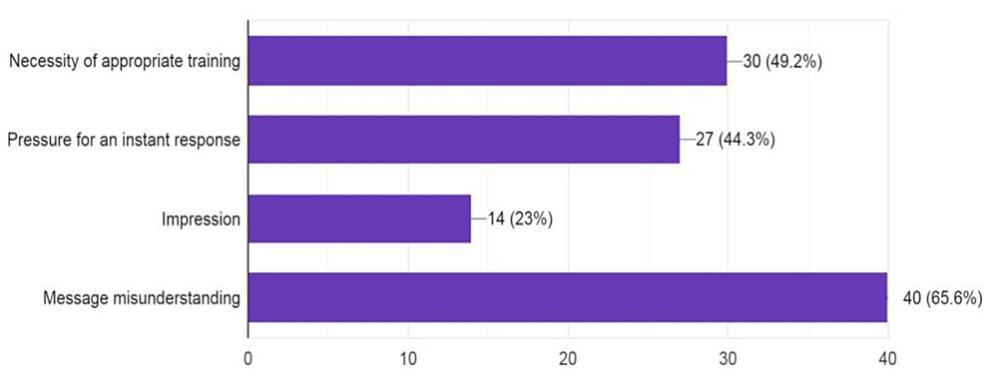
Potential shortcomings

The results regarding knowledge are shown in Table 2, Figure 1, and Figure 2. First, 88.5% of respondents were aware of teledentistry, and 11.5% were unaware and hence were excluded from the study. Second, 52.5% used SMS, WhatsApp, and email; 44.3% used audiovisual aids; and 3.2% used only verbal explanations in an audio aid method. Third, 67.2% of respondents did not know whether there are any guidelines given by the Dental Council of India to be followed to practice teledentistry; 18% responded yes and 14.8% responded no. Both explicit and implicit consent was necessary according to 62.3% of respondents. Also, 27.9% of respondents stated that only implicit consent was necessary, and 9.8% stated only explicit consent.

**Table 2:** Knowledge of participants regarding teledentistry DCI: Dental Council of India

Questions regarding knowledge of teledentistry	Percentage of responses		
	Yes	No	Don't know
Have you heard about teledentistry?	88.5%	11.5%	
Are there guidelines given by DCI to practice teledentistry?	18%	14.8%	67.2%
Is documentation important in teledentistry?	88.5%	11.5%	
Is preprinted consent useful?	68.9%	9.8%	21.3%
What are the media of teledentistry?	Audio aids (3.2%)	Audiovisual aids (44.3%)	Messenger (52.5%)
What type of consent should be taken?	Implied consent (27.9%)	Explicit consent (9.8%)	Both (62.3%)

Further, 88.5% of respondents stated that documentation was important, and 11.5% stated it was not important. Regarding knowledge of preprinted forms questions, 68.9% of respondents said preprinted forms were useful, 9.8% were not aware of it, and 21.3% said it was not useful. In the question related to the benefits of teledentistry, 67.2% and 57.4% of responses revealed that speed and low cost were the major benefits, respectively. Also, 52.5% noted that one benefit was documentation, and 41% noted efficacy. Additionally, 65.6% revealed that potential shortcomings were message misunderstanding followed by the necessity of appropriate training (49.2%), pressure for instant response (44.3%), and impression (23%).

**Figure 3:**
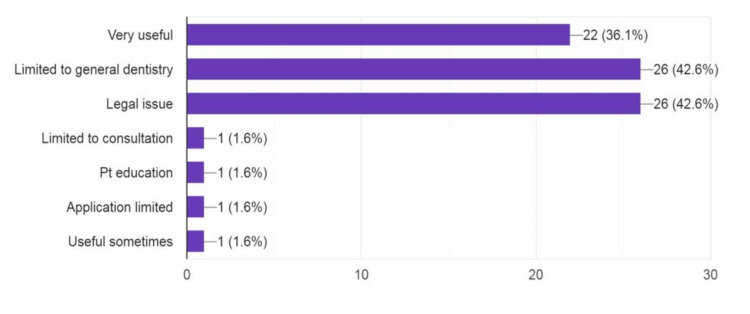
What is your opinion about teledentistry?

The results related to the attitude of dental professionals are shown in Figure 3 and Table 3. First, 93.4% responded that consent is important in dentistry, and 6.6% responded that it was not important. Second, 42.6% responded that they are concerned about legal issues and are limited to general dentistry. Third, 72.1% of respondents revealed that the doctor-patient relationship and a good level of surveillance are important, and 27.9% disagreed. For the question based on whether teledentistry will be a good tool for oral hygiene training, 50.8% agreed, 31.1 % strongly agreed, and 13.1% had a neutral response. A full 49.2% responded that teledentistry can help public health education, and 29.5% strongly agreed.

**Table 3:** Attitude of dental professionals about teledentistry COVID-19: coronavirus disease 2019

Questions regarding attitude about teledentistry	Yes			No	
Is consent important in teledentistry?	93.4%			6.6%	
Does teledentistry maintain the doctor-patient relationship and a good level of surveillance?	72.1%			27.9%	
Questions regarding attitude about teledentistry	Strongly agree	Agree	Neutral	Disagree	Strongly disagree
In the present COVID-19 pandemic, will teledentistry be a good tool for oral hygiene training?	31.1%	50.8%	13.1%	1.6%	3.3%
Can teledentistry help in health public education?	29.5%	49.2%	14.8%	3.3%	3.3%
Can teledentistry be financially feasible?	16.4%	44.3%	27.9%	9.8%	1.6%
Can teledentistry be time-saving?	18%	57.4%	18%	0%	4.9%
Can teledentistry be a precursor to robotic dentistry?	14.8%	26.2%	37.7%	16.4%	4.9%
Can specialist treatment at a distance, without compromising the quality of care, be possible with teledentistry?	6.6%	29.5%	23%	31.1%	9.8%

Regarding the financial feasibility of teledentistry, 44.3% strongly agreed, and 27.9% gave a neutral response. On whether teledentistry can be time-saving, 57.4% and 18% agreed and strongly agreed, respectively. The maximum neutral response of 37.7% was obtained regarding the question of robotics in teledentistry, and 26.2% agreed that robotics were important in teledentistry. Interestingly, 31.1% disagreed with the statement that specialist treatment at a distance will not compromise the quality of care, and 29.5% agreed, whereas 23% gave a neutral response.

## Discussion

The potential of teledentistry should be explored because it is a novel and modern tool using digital technology. Today, because access to the internet and telephonic systems has increased across fields, there is an opportunity for the development and application of teledentistry. Hence, the questionnaire was used to assess dental professionals' knowledge of and attitude toward teledentistry in the Sangli district of Maharashtra.

There is a lack of knowledge regarding teledentistry among dentists as only 61 out of 100 participants responded appropriately to the knowledge-related questions. These 61 responses also showed basic knowledge and a typical attitude toward teledentistry. Most respondents were women, had more than 10 years of experience, and were practicing in an urban area, indicating that the modern facilities of internet and social media communication are well utilized in the urban locality.

The comparison of results in the literature indicated that knowledge about teledentistry differs by country, level of education, and years of experience [13-17]. Dental students were found to be less knowledgeable and enthusiastic about teledentistry than dental faculty or practicing dentists [16]. Years of experience had an inverse relationship with the level of knowledge about teledentistry [15]. Despite many studies showing that most dentists and dental students wished to practice teledentistry in the future [14,17], approximately 26-31.4% of respondents did not think that teledentistry was efficient in many respects [15-17]. The studies also highlighted some barriers to teledentistry implementation that included the high cost of technology, lack of available technology, lack of human resources [17], lack of computer skills among dentists, and need to perform manual work rather than online procedures [18]. Few responses (29.5%) agreed that specialty treatment was possible without any compromise on the quality of care. In Mathivanan et al., 73% of dentists agreed that specialty treatment among the rural population can be performed [19].

Common messenger systems like SMS, WhatsApp, and email were used by respondents indicating the importance of digital documentation of dental treatment. A question on knowledge regarding consent revealed that both explicit and implicit consent were important, along with preprinted consent forms. Most respondents were not aware that there were no guidelines for teledentistry given by the Dental Council of India. More than 60% of the respondents considered that the advantages of teledentistry were its speed and low cost. Its disadvantages were misunderstanding and the necessity for appropriate training to gain knowledge about teledentistry. In correspondence with Al-Khalifa and AlSheikh, 60-70% of respondents were not sure about the technical reliability, privacy, and diagnostic accuracy of teledentistry [20].

Responses revealed that consent and legal issues related to teledentistry need to be considered and are important. Most responses stated that teledentistry is limited in general dentistry. More than 70% said teledentistry could maintain good dentist-patient relationships with a good level of surveillance. Also, 50%, 49%, and 44% agreed that teledentistry can be a good tool for oral hygiene, mass education, and financial feasibility, respectively. In another study, 48% of respondents agreed that public health education is enhanced through teledentistry [21]. More than 50% of respondents said that teledentistry is time-saving. The respondents agree that robotics in teledentistry is minimally important. A study by Abbas et al. concluded that most of the respondents agreed that teledentistry can improve healthcare (88.20%), enable access to rural patients (82.90%), and help save time [22]. Half of the general dentists had a positive attitude toward observing patients' dental problems over the internet and positively accepted teledentistry [23,24].

Most of the respondents agreed that teledentistry can be more convenient for oral examination and delivering healthcare. Around three-fourths of dentists believed that accurate information over telecommunication and specialist dental care can be provided to the rural population through teledentistry [25-27]. Most dentists agreed that teledentistry can reduce treatment costs and save time for both patients and dentists [28,29]. Around 90% of dental professionals believed teledentistry can invade the patient's privacy [30]. Although teledentistry has various shortcomings, it can be advantageous in developing a modern digitalized system in the field of dentistry.

## Conclusions

Although teledentistry was introduced early in 1997, it still remains relatively new and poses as an emerging branch of dentistry. Much is yet to be done to enlighten dental practitioners regarding the use and accessibility of this branch. The availability of such advanced digital technology is also a matter of concern, especially in developing countries like India. There is no doubt that if the existing barriers are addressed, then teledentistry can do marvels in revolutionizing both accessibility and time efficiency in dentistry, irrespective of specialization.
